# Studying visual search without an eye tracker: an assessment of artificial foveation

**DOI:** 10.1186/s41235-021-00304-2

**Published:** 2021-06-25

**Authors:** Laura E. Matzen, Mallory C. Stites, Zoe. N. Gastelum

**Affiliations:** Sandia National Laboratories, Mail Stop 1327, P.O. Box 5800, Albuquerque, NM 87185-1327 USA

**Keywords:** Eye tracking, Visual search, Artificial foveation

## Abstract

Eye tracking is a useful tool for studying human cognition, both in the laboratory and in real-world applications. However, there are cases in which eye tracking is not possible, such as in high-security environments where recording devices cannot be introduced. After facing this challenge in our own work, we sought to test the effectiveness of using artificial foveation as an alternative to eye tracking for studying visual search performance. Two groups of participants completed the same list comparison task, which was a computer-based task designed to mimic an inventory verification process that is commonly performed by international nuclear safeguards inspectors. We manipulated the way in which the items on the inventory list were ordered and color coded. For the eye tracking group, an eye tracker was used to assess the order in which participants viewed the items and the number of fixations per trial in each list condition. For the artificial foveation group, the items were covered with a blurry mask except when participants moused over them. We tracked the order in which participants viewed the items by moving their mouse and the number of items viewed per trial in each list condition. We observed the same overall pattern of performance for the various list display conditions, regardless of the method. However, participants were much slower to complete the task when using artificial foveation and had more variability in their accuracy. Our results indicate that the artificial foveation method can reveal the same pattern of differences across conditions as eye tracking, but it can also impact participants’ task performance.

**Supplementary Information:**

The online version contains supplementary material available at 10.1186/s41235-021-00304-2.

## Significance statement

There are numerous high-consequence activities that involve visual search. Pathologists look for abnormal cells in tissue samples, aviation security personnel search for potential threats in x-ray images of luggage, and imagery analysts search radar images for evidence of improvised explosive devices in war zones, just to name a few examples. Eye tracking is an extremely useful tool for studying how people perform these complex visual search tasks, often providing a much richer and more accurate picture of what the searcher is doing than can be obtained from their verbal report (Hayhoe et al., 1998). The information obtained from eye tracking experiments can be used to improve training, system design, and algorithms that support human decision making (cf. Matzen et al., 2016; Poole & Ball, 2006). However, there are many settings in which eye trackers cannot be used, either due to security restrictions, privacy concerns, funding limitations, or restrictions on in-person data collection. In this study, we sought to test the viability of artificial foveation as an alternative to eye tracking. If artificial foveation can reveal the same kinds of patterns in task performance that are revealed with traditional eye tracking methods, it could be a useful tool for studying visual search performance in situations where eye tracking is not possible, which is the case for many visual cognition researchers during the ongoing COVID-19 pandemic.

## Introduction

Eye tracking is a widely used tool for studying patterns of human attention (Holmqvist et al., 2011). In the human eye, the center of the field of vision, the fovea, has the highest visual acuity (Provis et al., 1998). We move our eyes to obtain high-resolution information from different parts of our visual environment (Henderson, 2003). These movements are called saccades, and the pauses between saccades, when the eyes are relatively stationary, are called fixations. Eye tracking studies are often based on the “eye-mind hypothesis,” which is the assumption that people fixate on the information to which they are attending (Just & Carpenter, 1976, 1984). Saccades are typically preceded by a shift in attention to a new location (Gottlieb et al., 1998; Kowler et al., 1995), so the pattern of saccades and fixations can be used to track shifts in attention as cognitive processing unfolds over time. Although humans can attend to things that they are not fixating with foveal vision, such as attending to something in peripheral vision or attending to auditory rather than visual inputs (Holmqvist et al., 2011; Underwood & Everatt, 1992), patterns of fixations are generally a reasonable indicator of what people are attending (Corbetta, 1998; Hayhoe, 2004; Just & Carpenter, 1980; Liversedge & Findlay, 2000; Rayner, 1998). Outside of laboratory experiments where participants are explicitly directed to attend to stimuli without fixating on them, there are few tasks where people would fixate on something other than the item that they are attending to (cf. König et al., 2016; Land, 2009; Land et al., 1999).

Since eye movements are a reasonable proxy for patterns of attention in most cases, recordings of eye movements can be a useful tool for studying human cognition. Eye trackers have become increasingly affordable and accessible, making them a popular tool for many types of research. In addition to studying cognition in laboratory settings, eye trackers are being used to study driving, marketing techniques, usability and user experience, web design, and numerous other applications (Poole & Ball, 2006).

While eye tracking can be a useful tool, there are also cases in which it is not practical. In naturalistic environments, where the experimenter has little control over what the participant does or sees, the data can be very difficult to analyze. In other settings, such as high-security environments, it may not be feasible to use an eye tracker due to concerns about the data collected by the eye tracker’s cameras. Additionally, in many applied research projects, the sponsors of the research may feel that the value added by using eye tracking is not worth the additional costs. After facing some of these challenges in our own work, we sought to test the effectiveness of using artificial foveation as an alternative to eye tracking in cases where we want to understand participants’ patterns of attention but are unable to use an eye tracker. In this paper, we describe our method for implementing this technique and compare the results to eye tracking data obtained from a visual search task.

## Artificial foveation

The term “artificial foveation” is most often used in the computer vision literature, when algorithms are developed to mimic properties of the human visual system. Computer vision researchers have found that using techniques which mimic foveal vision and eye movements have similar object detection performance as techniques that process the whole scene at high resolution, but with significantly less computational cost (Akbas & Eckstein, 2017). Other researchers have found that deep neural networks that are trained using images that are blurred to mimic human foveal and peripheral vision performed better on an object recognition task than models trained on other types of blur profiles (Pramod et al., 2018). Additionally, a deep learning approach to visual search that could change its sampling layout developed a sampling approach that resembles foveal vision (Cheung et al., 2016).

In our case, we are using the term “artificial foveation” to refer to an experimental technique that mimics foveal vision by blurring regions that the participant is not attending to. This approach is rooted in the history of process tracking research, which has developed various tools aimed at understanding how people acquire, integrate, and evaluate information when making decisions (Schulte-Mecklenbeck et al., 2011a). Eye tracking is often used as a process tracking tool, but there have been numerous efforts to develop other techniques that have lower cost or allow for data collection online rather than in a lab (Schulte-Mecklenbeck et al., 2011b).

One such method is mouse tracking. Software tools that stream the coordinates of a computer mouse while participants perform decision making tasks have allowed experimenters to test the temporal dynamics of decision making under different experimental conditions (Freeman & Ambady, 2010; Freeman et al., 2008; Spivey et al., 2005, 2008). Other researchers have used mouse tracking to create artificial foveation paradigms. In this case, the area of the screen immediately surrounding the mouse pointer is displayed at full resolution, while the rest of the display is blurred (Schulte-Mecklenbeck et al., 2011b). Alternatively, the relevant information may be covered by boxes, which open to reveal the information when the participant mouses over them (Jasper & Shapiro, 2002; Johnson et al., 2002; Willemsen & Johnson, 2010). Another approach requires participants to click the mouse to reveal high-resolution visual information in that region (Kim et al., 2017). All of these methods force participants to move the mouse around the screen to find and view the information that is relevant to the task they are trying to complete.

There are several mouse tracking tools that have been marketed to user experience (UX) and web designers, some of which explicitly promote themselves as an alternative to eye tracking. However, there has been little research comparing these approaches to standard eye tracking methods. One commercially available tool, Attensee, provides a comparison to data collected from a Tobii eye tracker by showing heatmaps generated from the eye tracking data and from their mouse tracking method side-by-side (http://www.attensee.com/casestudies/tobii/). While the heatmaps look similar, no quantitative comparison is provided, and neither heatmap provides any information about the dynamics of the viewers’ attention (e.g., the time spent viewing the image, the order in which the different regions were viewed, etc.).

Only a handful of studies have directly compared artificial foveation methods implemented via mouse tracking to data collected via eye tracking. The researchers who developed the Flashlight tool (Schulte-Mecklenbeck et al., 2011b) compared their method to eye tracking on three tasks: an arithmetic task, a gambling task, and a reading task. They compared participants’ accuracy, completion time, fixations, and pattern and sequence of information acquisition across both methodologies in a between-subjects design. They found no significant differences between the two methods in terms of participants’ accuracy, but participants had more fixations on all of the tasks in the eye tracking condition and longer response times for two of the three tasks in the Flashlight condition. The patterns of fixations were compared via heatmaps and transition matrices and were visually similar to one another.

In another study, heatmaps obtained through the use of the BubbleView tool (Kim et al., 2017) were compared to eye tracking data for free viewing and description tasks involving different types of visual stimuli (information visualizations, natural images, and static webpages). The researchers found that the distribution of clicks in the BubbleView tool were a reasonable approximation of the distribution of fixations collected via eye tracking. Similarly, Jiang et al. (2015) developed an artificial foveation technique based on mouse tracking. Participants moved the mouse to explore images of natural scenes in a free viewing task. The artificial foveation data were collected both in a laboratory setting and online, via Amazon Mechanical Turk. The researchers found that the distributions of mouse movements from both data collection scenarios were similar to the distribution of fixations obtained via eye tracking for participants viewing the same images.

The results of these studies suggest that artificial foveation techniques based on mouse tracking could be a useful proxy for eye tracking in situations where using an eye tracker is not feasible. However, due to the scarcity of studies in this area, there are numerous unanswered questions. For example, is artificial foveation effective in visual search paradigms, where participants are using visual information to find some target item of interest? Visual search is one of the cognitive processes that it most commonly studied using eye tracking, yet, to our knowledge, none of the prior studies on artificial foveation techniques have incorporated a visual search task. Instead, they have used tasks involving open-ended exploration (Egner et al., 2018; Jiang et al., 2015; Kim et al., 2017) or tasks that required access to visual stimuli but were not primarily visual tasks from the perspective of cognitive processing (Schulte-Mecklenbeck et al., 2011b).

Our study aimed to address this gap by performing a direct comparison between eye tracking and artificial foveation for a visual search task. Our artificial foveation method was implemented in E-Prime 3.0 software (Psychology Software Tools, Pittsburgh, PA), a widely used software package for stimulus presentation. Our code is available in Additional file 1 so that other researchers can use our approach to implement their own artificial foveation experiments in E-Prime. Our approach is a hybrid of the techniques that have been used in prior work. Like the BubbleView (Kim et al., 2017) and Flashlight (Schulte-Mecklenbeck et al., 2011b) tools, participants are able to see a blurred version of the entire stimulus, allowing them to get an overview of the locations of different pieces of information. However, instead of showing a circle of high-resolution information around the mouse pointer, we covered each item on the screen with a mask that is removed when the mouse pointer is in the vicinity of that item. This approach is similar to the box-based method of revealing information that has been used in decision making studies (Jasper & Shapiro, 2002; Johnson et al., 2002; Willemsen & Johnson, 2010). One notable difference is that our display had 72 boxes, rather than the small number of boxes that has typically been used in decision making studies. Since our focus is on visual search, the large number of items ensured that we could collect sufficient data on each trial to assess the impact of different search constraints on the participants’ performance and search strategies.

Our implementation of artificial foveation shares some similarities with the gaze-contingent paradigms pioneered by Rayner and colleagues, such as the moving window, moving mask, or boundary change paradigms (for reviews, see Rayner, 1998, 2009, 2014; Schotter et al., 2012). These techniques have traditionally been employed to study the basic characteristics of foveal vision and the relationship between foveal and parafoveal processing in skilled reading. In the gaze-contingent moving window paradigm (McConkie & Rayner, 1975), an invisible “window” is created around the reader’s fixation point; the text shown within the window is correct, but the text outside of the window is manipulated in some way. The size of the window may be adjusted to measure how much information readers need to see in central fixation in order to maintain normal reading patterns. The text outside of the window may also be manipulated (i.e., replaced with x’s, similar looking letters, or filling in spaces) in order to understand how readers use parafoveal information to plan eye movements. In general, this research has shown that as long as the letters in foveal vision are correct (the central 2°, usually 6–7 letters in lab-based experiments), and that the letters in the perceptual span are maintained (usually 3–4 letters to the left of fixation and 14–15 characters to the right of fixation, in English), reading proceeds more or less normally (for reviews, see Rayner, 2009; Schotter et al., 2012). Our implementation of the artificial foveation paradigm most closely parallels the moving-window paradigm with a 13-character window, which is just under the typical reader’s perceptual span and should allow reading of our experimental items to be relatively unimpeded by the window.

Our task involved a list-to-list comparison in which participants had to check off all of the items on one list by comparing them against a second list. The task was inspired by the cognitive demands on inspectors in the international nuclear safeguards domain (Gastelum et al., 2017). Inspectors working for the International Atomic Energy Agency (IAEA) conduct inspections of nuclear facilities to ensure that nuclear materials are not being diverted from known, safeguarded facilities and that those facilities are not being misused for undeclared purposes. They must complete complex visual search tasks in which they compare a facility’s declarations to its inventory records, physical inventory, and reports from prior inspections. Although safeguards inspectors do not have control over the formatting of the materials provided by the facility, they do have control over the inspection-related materials that they bring with them into the field, such as inventory records from prior inspections. In a series of experiments, we tested whether changes to the formatting of an inspector’s checklist would support faster and more accurate inspection of a facility’s records and inventory (Gastelum et al., 2018; Matzen et al., 2019). We manipulated the presentation of the “inspector’s” list by changing the ordering of the information and by providing color-coded cues that could be used to constrain the visual search process. These experiments showed that changes to the formatting of the inspector’s list impacted participants’ speed, accuracy, and likelihood of missing subtle errors for several different variants of the inspection task. In particular, we found that participants made good use of color cues that indicated which column of the facility’s inventory list was most likely to contain each item from their checklist. The participants used those cues to constrain their visual search process to the appropriate column, allowing them to complete each trial and check off the whole list much faster than they could without the assistance of the color cues. Other list presentation methods that could have constrained their visual search process, such as organizing the checklist in numerical order, were less helpful. The participants could have selected items from the facility’s list and used the seal number to narrow in on where to find the corresponding item in their checklist. However, very few participants adopted this strategy, preferring to select an item from their checklist to search for in the facility’s list, even when searching in the opposite direction would have been much faster.

In order to assess the effectiveness of artificial foveation as a technique for studying visual search, we selected one of the experiments from our prior work and implemented it as an artificial foveation task instead of an eye tracking task. We collected data from a new group of participants under the artificial foveation paradigm and compared the results to the results of the eye tracking experiment. We assessed the effectiveness of the artificial foveation approach by comparing it to eye tracking in terms of the participants’ behavioral performance, response times, number of fixations, and search strategies.

## Methods

### Participants

The participants in both experiments were recruited from the employee population of Sandia National Laboratories and were compensated for their time. The studies were reviewed and approved by Sandia’s Human Studies Board.

The eye tracking study had 18 participants, three of whom were later excluded from the analysis due to failure to follow the task instructions (two participants) or poor eye tracking data (one participant). The remaining 15 participants (11 males and 4 females) had an average age of 37 years. The data collected from these participants has been previously published (Gastelum et al., 2018).

To compare our artificial foveation method to eye tracking, we recruited an additional 15 participants (8 males and 7 females) for the artificial foveation task. The participants had an average age of 35 years.

### Task and materials

Both groups of participants completed the same task, which involved comparing two lists of alphanumeric strings. The task was designed to mimic an inventory checking task (as described in Gastelum et al., 2018), where one list of sealed container numbers is checked against another. The participants completed the list checking task a total of six times using six different pairs of lists. The items on the “facility’s list” were always presented in a random order. The items on the “inspector’s checklist” were formatted differently in each of the six conditions, as described below.

Prior to beginning the list checking task, the participants were given some background information about the international nuclear safeguards domain and were told that they would be acting as facility inspectors, comparing their inspection checklists against the “facility’s” inventory lists. Their instructions were as follows: “In this experiment, you will complete a visual inspection task in which you will be comparing one list to another. The lists will be organized in different ways, and we are testing which type of organization leads to the best performance in terms of the speed and accuracy of the inspection. This task is based on tasks that are common for inspectors from the International Atomic Energy Agency (IAEA). These inspectors visit nuclear facilities to ensure that all nuclear materials are accounted for. For example, they may apply seals to containers of nuclear materials to ensure that none of the material can be removed. On subsequent visits, they will check their records of the container and seal numbers against the inventory records provided by the nuclear facility. In this experiment, you will be conducting a simulated version of this task. In this experiment, the facility's inventory list will always be presented on the right side of the computer screen. This list will always look the same. On the left side of the screen, you will see your list. You will act as the inspector and check to see if the seal numbers on your list match the seal numbers on the facility's list. You will also check to make sure that the seals are listed with the correct container number. You will check off the items on your list one by one until you have completed your inspection. The seal numbers are always a six digit number, like 324159. The container numbers have four characters, like ZG-91.”

**Fig. 1:**
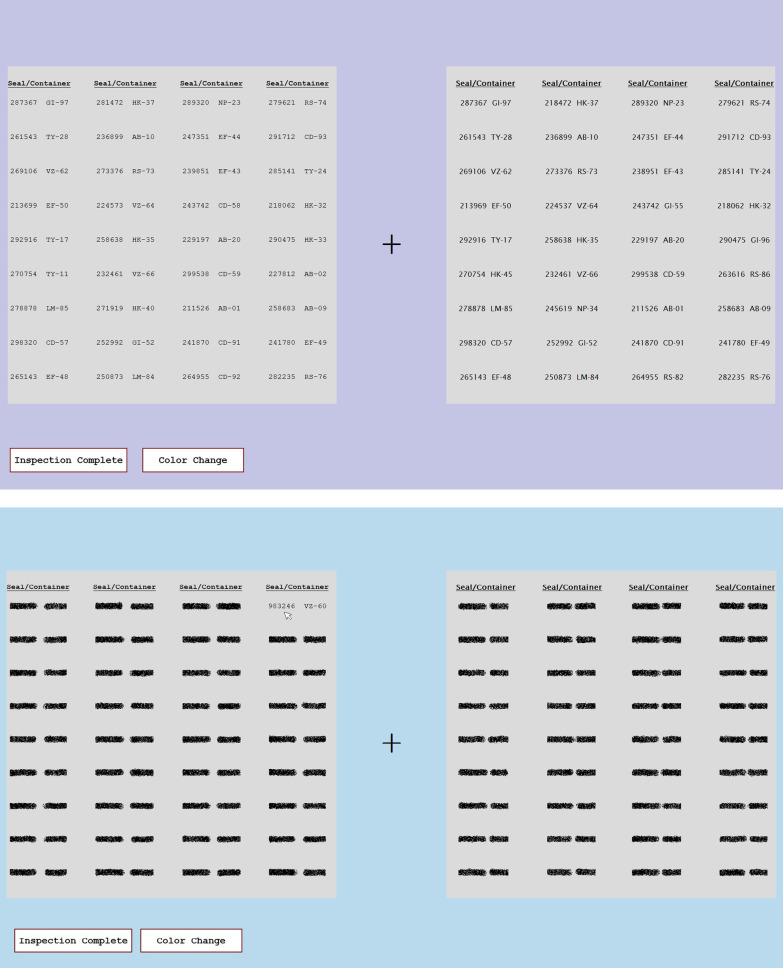
Examples of the stimulus display for the eye tracking (top) and artificial foveation (bottom) experiments. These screen shots show how the stimuli appeared to the participants in each experiment

Figure 1 shows screen shots of the display shown to the participants during the list checking tasks. The top panel shows the eye tracking version of the task, in which all of the items were shown in high resolution. The bottom panel shows the artificial foveation version of the task, in which all of the items were obscured by a visual mask until the participant hovered the mouse over them. In this example, the participant’s mouse is revealing the top item in the fourth column.

Both the inspector’s checklist (left) and the facility’s inventory list (right) had 36 seal-container pairs, arranged in nine rows and four columns. The seal-container pairs were shown in 14-point Courier New font on the inspector’s list and in 14-point Lucida Sans font on the facility’s list. The two fonts were used so that the items on the two lists were not perceptually identical to one another. On both lists, the items were spaced so that there was a buffer 70 pixels wide between each seal-container pair and its neighbors in the same row or column. In our experimental setup, this was equivalent to approximately 1.4° of visual angle. This spacing ensured that the participants could not easily read more than one item at a time and that fixations were assigned to the correct item in the eye tracking experiment.

The items on the lists, each of which represented a seal-container pair, were 140 pixels wide (approximately 2.8° of visual angle) and 15 pixels tall (approximately 0.3° of visual angle). The seal numbers were six-digit numerical strings such as “945384” and the container names consisted of two letters and two numbers, separated by a hyphen, such as “AB-37.” There were two spaces between the seal number and the container number in each pair. All of the seal-container pairs in each list were unique, but they were controlled in certain ways to make the visual search task more difficult. First, within each list, all of the seal numbers started with the same digit. To avoid any patterns within the seal numbers that could have attracted participants’ attention or made some numbers more memorable than others, the final five digits were pseudorandomly generated such that every digit (0–9) appeared approximately the same number of times in each position. Second, the letters within the container names were repeated across different containers. There were ten possible letter pairs, each consisting of two unique letters (AB, CD, EF, HK, LM, NP, RS, TY and VZ). The same letter pairs were repeated multiple times in each list, but always with different numbers attached such that each container number was unique.

The 36 seal-container pairs on each inspector’s list fell into one of five conditions. Eighteen “Match” items were matches to pairs on the facility’s list. Four of items on the inspector’s list, the “Wrong Container” items, contained seals that were on the facility’s list but paired with a different container number. Two “Missing” items on the inspector’s list were entirely missing from the facility’s list. Four of the seal numbers on the inspector’s list, the “Transposed” items, had a near-match on the facility’s list, but with two numbers transposed. For example, the seal-container pair on the inspector’s list might be “239851 EF-43” while the corresponding item on the facility’s list was “238951 EF-43”. Finally, eight of the items on the inspector’s list had both a match on the facility’s list and a transposed version of the seal that appeared on both lists. For example, seal “261543” and seal “265143” both appeared on the inspector’s list and both had a match on the facility’s list. These were called the “Transposed Match” items and were included to increase the difficulty of the matching task. Although both of these similar seal numbers had a match on the facility’s list, participants who were not paying close attention might check off the wrong one, which could lead to mismatches later in their inspection process.

To complete the task, the participants had to compare the inspector’s list to the facility’s list. Each time they found one of the items from their list in the facility’s inventory, they clicked on that item on their list. Clicking on an item brought up four response choices in the center of the screen: “Seal present, correct container,” “Seal present, incorrect container,” “Seal missing” and “Other issue.” Participants clicked on one of the four responses, and then the corresponding item was greyed out on their list, indicating that it had been checked off. The first response choice, “Seal present, correct container” was the correct response for 26 of the items on the list, the Match and Transposed Match items. The response “Seal present, incorrect container” was the correct response for the four Wrong Container items. “Seal missing” was the correct response for the Missing items. For the Transposed items, either “Seal missing” or “Other issue” was counted as a correct response.

After checking off all the items in their list, the participants clicked on the “Inspection Complete” button to indicate that they had finished the task.

Participants completed the inspection task six times, with six different lists that comprised a 3 × 2 within-subjects design. The seal-container pairs in the facility’s list were always presented in a random order, but the presentation of the participant’s checklist was manipulated across conditions. There were three ordering conditions for the participant’s list: Random Order, Numerical Order by seal number, or Facility Order (in which the seals were presented in the same order as those in the facility’s list). There were also two color-coding conditions. In three of the lists (one in each of the list order conditions), all the list items were presented in black font. In the other three, the items on the participant’s list were color-coded according to which column of the facility’s list contained the corresponding item.

**Fig. 2:**
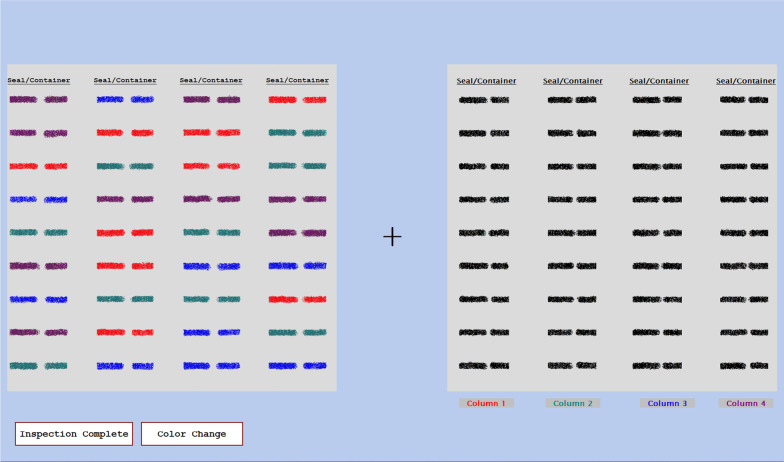
An example of a participant’s color-coded list from the artificial foveation experiment

The color coding and list ordering conditions were intended to aid the participants by constraining their search process. When their list was in the same order as the facility list, the participants generally knew exactly where to look in order to compare the seals on the two lists. There were errors, such as the Transposed, Wrong Container, and Missing items, so there was not a perfect match between the two lists, but the participants could easily check off most of the items on their list simply by looking at the corresponding position on the facility’s list. Figure 1 shows an example of the Facility Order, No Color condition. Figure 2 shows an example of the Random Order, Color Coded condition in the artificial foveation version of the experiment. For any of the color coding conditions, the colors of the items on the inspector’s list corresponded to one of the columns on the facility list, allowing participants to narrow their search to only one column. This condition mimicked real-world scenarios in which different containers might be stored in different locations in a facility, and thus grouped into smaller subsets on the inventory list. In the color conditions, participants typically only needed to look at nine of the 36 seals on the facility’s list. In the case of a Missing seal, they might search the additional columns as well, to ensure that the seal truly was missing from the facility’s list. Figures 9 and 10 show additional examples of lists with color coding cues. Figure 9 shows a list from the Numerical Order, Color Coded condition and Fig. 10 shows a list from the Random Order, Color Coded condition.

In the Numerical Order condition, participants could constrain their search if they started from the facility’s list and then used the numerical ordering of the inspector’s list to quickly zero in on the correct seal (or seals, in the case of the Transposed Match items, which were typically close together on the list in this condition) to check. The Random Order, No Color condition (also shown in Fig. 10) was expected to be the most difficult, since the inspector’s list did not provide any cues about where to look in the facility’s list, or vice versa. Thus, the participants had to search through all 36 items on the facility’s list until they found the item they were looking for.

While this experiment differed in many ways from classic visual search tasks, such as identifying a *T* in an image full of *T* and *L* stimuli (e.g., Wolfe, 1998; Wolfe et al., 1989), the list presentation manipulations led to different set sizes in the visual search task. In the Random Order, No Color condition, the set size was all 36 items. In the Color Coding conditions, the effective set size was nine items. And in the Facility Order condition, the set size was one item, since participants knew exactly where to look for each seal-container pair.

Prior to starting each of the six blocks, the participants were told how the inspector’s list in the upcoming block would be organized. The explanation of the list formatting stated whether or not there was color coding corresponding to the columns in the facility’s list and whether the inspector’s list would be shown in random order, numerical order, or the same order as the facility list. There were no suggestions about how to use this information and participants were free to adopt their own strategies for completing each checklist.

### Secondary change detection task

While participants were completing the inspection task, the background color of the screen changed occasionally. The images in Figs. 1 and 2 show the three possible background colors. The background color changed either two or three times during each inspection task. The changes were linked to particular seals, such that after a participant clicked on that seal, the background color would change on the next trial. The seals that triggered the color changes were different for each block. Participants were instructed to click on a button labeled “Color Change” as soon as they noticed a change in the background color. At the end of each block, they were also asked to report how many times the color had changed during that inspection task. Their choices ranged from zero to four, and they clicked on the number corresponding to their answer. The color change detection task was included as a measure of the participants’ situational awareness during the inspection task.

### Eye tracking procedure

After giving their informed consent, participants were seated in a dimly lit, sound attenuating booth. They were seated so that their eyes were approximately 80 cm from the computer monitor, which was 52 × 32 cm in size. The monitor displayed the stimuli at a resolution of 1920 × 1080 pixels. Participants completed a practice session that explained the task, including the background color changes and all of the possible configurations of the inspector’s checklist. Then they completed a simple version of the inspection task in which they had to check off a list of four seals. After participants had completed the practice block and indicated that they understood the task, the eye tracker was calibrated. Eye tracking data was collected with a Fovio eye tracker and recorded and analyzed with EyeWorks software. The participants completed a five-point calibration sequence, and then the accuracy of the calibration was assessed by the experimenter and repeated if necessary. The calibration process was repeated prior to each block of the experiment.

The practice and task blocks were presented using E-Prime 3.0 software. The participants completed the six blocks of the experiment in a random order. Each block began with a description of how the inspector’s list would be organized. Each trial began with a fixation cross that was presented in the center of the screen for 1.5 s. Then the lists appeared on the screen and remained there until the participant clicked on a seal on the inspector’s list. Clicking on a seal added the four response choices to the screen. After the participant clicked on one of the response choices, the next trial began and the seal that had been checked off turned silver, indicating that the response had been recorded. Clicking on the “Color Change” button also initiated a new trial. Once participants had checked off all of the seals on the inspector’s list, they clicked the “Inspection Complete” button. They were then asked to indicate how many times the background color changed during the inspection task. Finally, participants were asked to give a brief description of the strategy that they used during the inspection task.

### Artificial foveation procedure

**Fig. 3:**
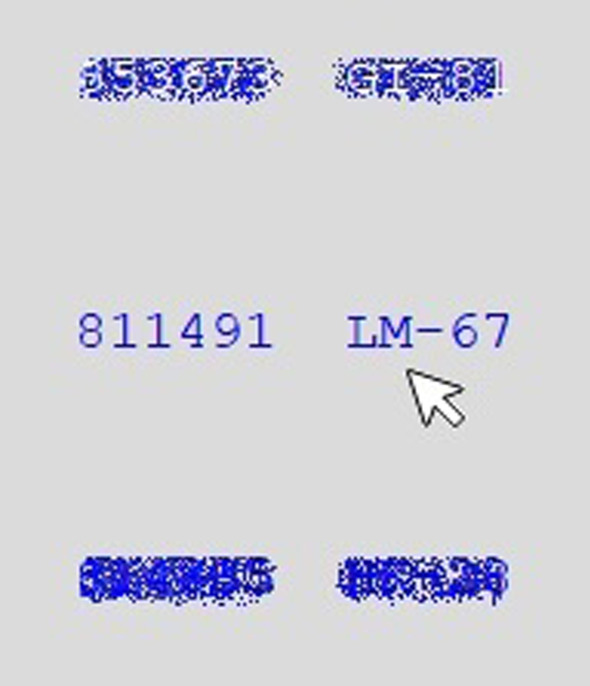
An example of an item that has already been checked off of the participant’s list (top), an item that the participant is currently viewing by pointing to it with the mouse (center), and an item that is masked and not yet checked off of the participant’s list (bottom)

The study environment for the artificial foveation experiment was identical to that of the eye tracking study, except that the eye tracker was not used. Participants were seated in a dimly lit, sound attenuating booth with their eyes approximately 80 cm from the computer monitor, which was 52 × 32 cm in size and displayed the stimuli at a resolution of 1920 × 1080 pixels. The participants were given a small lap desk on which they moved the mouse. They completed the same list inspection task, but in this case the E-Prime experiment files were constructed such that the seal-container pairs were masked until a participant moved his or her mouse into the area around one of the items. The mask image was *behind* the text of seal-container pairs but matched them in color, making them impossible to read. E-Prime was used to track the mouse movements and to update the display based on the position of the mouse. There were 72 regions defined on the screen, one for each of the seal-container pairs in both lists. Each region was 144 pixels wide and 20 pixels tall, which accommodated the seal-container text plus a buffer zone around it. Any time the mouse entered one of those regions, an image of the appropriate seal-container pair surrounded by a grey box was added to the display in that location. This gave the appearance of removing the visual mask from that item. When the mouse left that region, the superimposed text box disappeared, showing the visual mask once again. When participants checked off one of the items on their list, the color of the text for that item changed to white. The white text was visible in front of the visual mask, allowing participants to see which items had been checked off of their list, just as they were able to see them in the eye tracking study. Figure 3 shows an example from one of the color-coded conditions. The mouse is in the region near the center item, so the mask for that item is hidden and the text is visible. The top item has already been checked off the list, so the text appears in white. It is difficult to read the text without mousing over that region to remove the mask, but participants can tell that the item there has already been checked off. The bottom item has not yet been checked off, so it appears as blue text on top of a blue mask.

## Results

### Accuracy

The participants’ accuracy for each list was calculated by determining how many of the 36 seal-container pairs on their inspection checklist was categorized correctly (as a match, missing, etc.). In general, participants performed near ceiling on both the eye tracking and artificial foveation tasks. The mean accuracy and the standard deviation for each list presentation condition in both experiments is shown in Table 1. A 2 × 3 × 2 ANOVA (experiment type × list order condition × color coding condition) showed that there was not a significant difference in accuracy between the two experiments (*F*(1,140) = 1.81, *p* = 0.19, *η*_p_^2^ = 0.06). There was a significant effect of color coding (*F*(1,140) = 5.85, *p* < 0.02, *η*_p_^2^ = 0.02), where accuracy was significantly higher for lists that had color coding than for those that did not. There was not a significant effect of list ordering condition (*F*(2,140) = 1.12, *p* = 0.33, *η*_p_^2^ = 0.01) and there were no significant interactions (all *F*s < 1.67, all *p*s > 0.19).

**Table 1:** Average accuracy (and standard deviation) for each list presentation condition for the eye tracking and artificial foveation experiments

	Facility order		Numerical order		Random order	
	Color	No color	Color	No color	Color	No color
Eye tracking	95.6% (4.0%)	95.0% (3.8%)	96.9% (3.1%)	95.3% (4.6%)	96.1% (4.3%)	94.4% (6.5%)
Artificial foveation	94.6% (7.0%)	94.0% (6.4%)	93.0% (10.8%)	92.0% (6.4%)	94.0% (5.1%)	90.0% (11.3%)

#### Change detection task accuracy

Participants in both experiments had a secondary task of indicating when the color of the background changed during the visual search task. Participants had equally poor performance on this task for both the eye tracking and artificial foveation experiments. The background color changed 2–3 times during each of the six blocks, depending on the order in which the participants clicked on specific items in the checklist. On average, participants in the eye tracking experiment detected 57.6% (SD = 36.0%) of the changes and participants in the artificial foveation experiment detected 59.9% (SD = 34.6%) of the changes. There was not a significant difference between the two groups (*t*(28) = 0.18).

#### Task completion strategies

To assess whether the constraints of the artificial foveation task impacted the way in which participants performed the task, we analyzed the order in which they checked off the items on the inspection list in both experiments. For the eye tracking experiment, as discussed in Gastelum et al. (2018), we identified five general patterns: checking off one row or column at a time, checking off one color group at a time, using numerical ordering to locate seals, or checking off items in a seemingly random order (“other”). Table 2 shows which strategies the participants used in each list presentation condition for each experiment. Recall that there were 15 participants in each experiment, so each half of the table shows a different set of 15 participants. In addition, note that in the condition where the inspection list was color coded and presented in the Facility Order, each column corresponded to one color in the color coding scheme, so checking off the items column-by-column is the same as checking them off color-by-color.

**Table 2:** The number of participants using each task completion strategy for each list presentation condition in the eye tracking and artificial foveation experiments

List condition		Eye tracking task completion pattern					Artificial foveation task completion pattern				
List order	Color coding	Rows	Columns	Color groups	Numerical order	Other	Rows	Columns	Color groups	Numerical order	Other
Facility order	Yes	2	13		N/A	0	0	15		N/A	0
	No	6	9	N/A	N/A	0	3	12	N/A	N/A	0
Numerical order	Yes	3	6	6	0	0	0	6	9	0	0
	No	3	8	N/A	4	0	1	9	N/A	4	1
Random order	Yes	4	6	5	N/A	1	0	5	10	N/A	0
	No	7	7	N/A	N/A	1	3	10	N/A	N/A	2

This comparison indicates that participants in the artificial foveation condition were more likely to use the color coding information to guide the order in which they checked off items from their list. Since the task was more tedious and time consuming in the artificial foveation experiment, the participants may have relied more heavily on this cue to organize their approach to the task. However, the participants were no more likely to use the numerical ordering to speed their search process, when it was available. Only a small number of participants (four in each experiment) made use of the numerical ordering.

### Response times

**Fig. 4:**
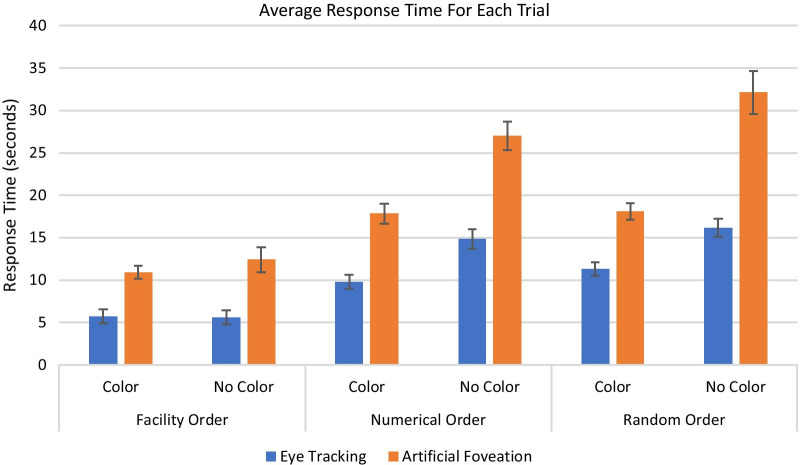
Average response time for each list presentation condition for the eye tracking and artificial foveation experiments. Error bars show the standard error of the mean

The participants’ response times were calculated for each trial based on the time from trial onset to the time they clicked on an item on their checklist to check it off. Their average response time per trial was calculated for each of the six list conditions. The results are shown in Fig. 4.

A 2 × 3 × 2 ANOVA (experiment type × list order condition × color coding condition) showed that there was a significant difference in response times between the two experiments (*F*(1,140) = 37.10, *p* < 0.001, *η*_p_^2^ = 0.21), with participants taking significantly longer to complete each trial in the artificial foveation task. For the eye tracking study, the average response time was 10.6 s (SD = 3.2 s), while the average response time was 18.7 s (SD = 4.0 s) for the artificial foveation study. There was also a significant main effect of list order (*F*(2,140) = 152.58, *p* < 0.001, *η*_p_^2^ = 0.69), and a significant main effect of color coding (*F*(1,140) = 108.36, *p* < 0.001, *η*_p_^2^ = 0.44). There was a significant three-way interaction between experiment type, list order, and color coding condition (*F*(2, 140) = 3.70, *p* < 0.03, *η*_p_^2^ = 0.05) and significant two-way interactions between experiment type and list order, experiment type and color coding, and list order and color coding (all *F*s > 7.14, all *p*s < 0.01).

Within each dataset (eye tracking or artificial foveation), we ran a 3 × 2 within subjects ANOVA to examine the interactions between the list ordering conditions and the color coding conditions. In both experiments, there were significant main effects of list order (eye tracking experiment: *F*(2, 70) = 157.54, *p* < 0.001, *η*_p_^2^ = 0.82; artificial foveation experiment: *F*(2, 70) = 72.80, *p* < 0.001, *η*_p_^2^ = 0.65), significant main effects of color coding (eye tracking experiment: *F*(1, 70) = 67.90, *p* < 0.001, *η*_p_^2^ = 0.49; artificial foveation experiment: *F*(1, 70) = 73.36, *p* < 0.001, *η*_p_^2^ = 0.47), and significant interactions (eye tracking experiment: *F*(2, 70) = 18.44, *p* < 0.001, *η*_p_^2^ = 0.35; artificial foveation experiment: *F*(2, 70) = 14.36, *p* < 0.001, *η*_p_^2^ = 0.26).

#### Impact of effective set size

While this task was not a traditional visual search task, we still had conditions that effectively had different set sizes, as described in the methods section. For the two facility order conditions, the effective set size was one, since participants only had to check one location in the facility list to confirm whether the corresponding item on their list was a match. For the numerical order + color coding condition and the random order + color coding condition, the effective set size was nine, since the participants could constrain their search to a single column of the facility’s list or items of a single color on the inspector’s list. For the random order condition without color coding, the participants had to search through up to 36 items on the facility’s list before finding the seal-container pair that they were looking for.

**Fig. 5:**
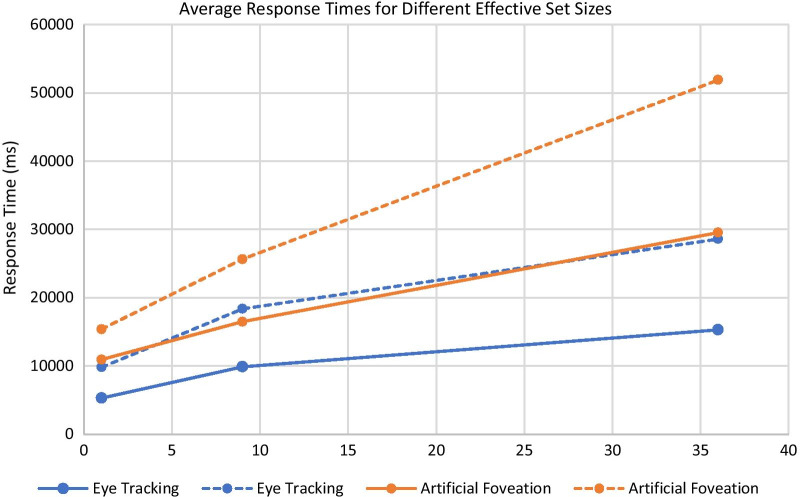
The average response times for the eye tracking (blue) and artificial foveation (orange) experiments when the effective set size for the search task was 1, 9, and 36. The solid lines show the trials in which participants checked off a Match item and the dashed lines show the trials in which participants checked off a Missing item

Figure 5 shows the participants’ average response times for each effective set size in the eye tracking and artificial foveation paradigms. The solid lines show the participants’ average RTs for match trials (trials when they checked off an item on their list that was a match to an item on the facility’s list) while the dotted lines show the participants’ average RTs for missing trials (trials where they checked off an item on their list that was missing from the facility’s list, and correctly identified it as being a missing item). As seen in classic visual search tasks such as the T and L task (Wolfe, 1998), the participants’ RTs increased roughly linearly as the effective set size increased.

In order to more directly compare our findings to standard set size effects in the visual search literature, individual set size slopes were calculated by computing linear regressions on RTs for each subject, for match trials and missing trials (i.e., target present and target absent) separately. Two participants from the artificial foveation experiment were removed from this analysis due to missing data in the “target missing” condition. A mixed ANOVA was conducted on the slope data with the between-subjects factor of experiment (eye tracking vs. artificial foveation) and the within-subjects factor of trial type (match vs. missing). Results showed a main effect of experiment (*F*(1, 26) = 20.62, *p* < 0.001, *η*_p_^2^ = 0.44), indicating that the slopes for the artificial foveation paradigm were larger overall. In addition, the slopes were larger for missing items than for match items (*F*(1, 26) = 48.56, *p* < 0.001, *η*_p_^2^ = 0.96). This pattern indicates that our task is comparable to the classic visual search tasks that require inefficient visual search. Importantly, there was no interaction between experiment and trial type (*F*(1, 26) = 1.69, *p* = 0.20). This indicates that although the slopes were larger overall for the artificial foveation experiment, the magnitude of the difference between slopes for match trials and missing trials was comparable for the two experiments. To confirm this, we compared the slope ratio between the match and missing trials (mean artificial foveation ratio = 1.74, mean eye tracking ratio = 2.08). A between-subjects ANOVA with the factor of experiment confirmed that these slope ratios were not significantly different (*F*(1, 26) = 1.44, *p* = 0.24). Together, these results show that the relationship between target present and target absent search slopes was consistent across the two methodologies. The artificial foveation paradigm slowed participants down in general, but did not change the dynamics of the visual search process.

### Visual search dynamics

To further investigate the relationship between the metrics produced by the artificial foveation paradigm and metrics that are commonly applied to eye tracking data, we compared how long, how often, and in what order the participants fixated on different types of items within the lists. First, we calculated the number of fixations (real or artificial) per trial to confirm the assumption that the more difficult list presentation conditions that produced longer response times per trial also had higher numbers of fixations per trial. We also assessed how often participants fixated and refixated on specific types of list items (Match, Wrong Container, etc.) within each trial. Next we used the eye tracking and artificial foveation data to compare how long the participants dwelled on the easier and more difficult list items during their visual search process. Finally, we used the artificial foveation data to generate scan paths that could be compared to scan paths produced by eye tracking data. We then used the scan paths to investigate whether or not the participants in the two experiments used similar search strategies despite the different constraints imposed by the artificial foveation paradigm.

#### Number of fixations per trial

**Fig. 6:**
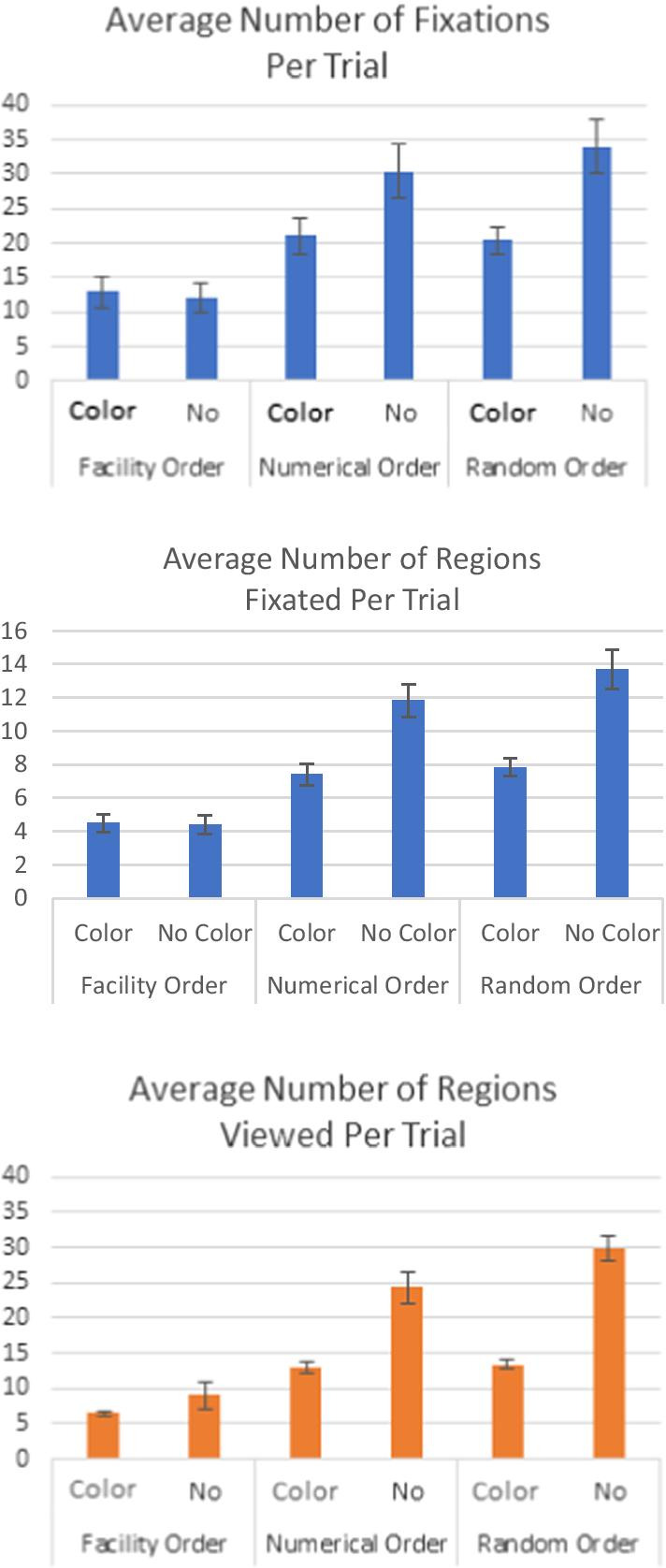
The average number of fixations per trial for the eye tracking experiment (top), the average number of regions fixated per trial for the eye tracking experiment (middle), and the average number of regions viewed per trial for the artificial foveation experiment (bottom). Error bars show the standard error of the mean

We compared the average number of regions viewed per trial in the artificial foveation paradigm to the average number of fixations per trial and the average number of regions fixated in the eye tracking paradigm. These results are shown in Fig. 6. The number of regions fixated per trial was lower in the eye tracking paradigm than in the artificial foveation paradigm, but the total number of fixations was higher in the eye tracking paradigm. This makes sense because it could take multiple fixations to read a seal-container pair, and these are counted separately within a single region for the eye tracking experiment, but the time it takes to read the seal-container pair only counts as one region view in our implementation of artificial foveation. Despite this difference, the patterns across conditions are remarkably similar for all three metrics. Once again, both data collection methods showed the same pattern of results. When looking at the *total* number of fixations per trial or the number of *regions* fixated per trial in the eye tracking experiment, there were significant main effects of list order (fixations per trial: *F*(2, 70) = 56.11, *p* < 0.001, *η*_p_^2^ = 0.62; number of regions fixated per trial: *F*(2, 70) = 92.78, *p* < 0.001, *η*_p_^2^ = 0.73) and color coding (fixations per trial: *F*(1, 70) = 34.07, *p* < 0.001, *η*_p_^2^ = 0.33; number of regions fixated per trial: *F*(1, 70) = 70.90, *p* < 0.001, *η*_p_^2^ = 0.50) and there was a significant interaction between the two (fixations per trial: *F*(2, 70) = 11.70, *p* < 0.001, *η*_p_^2^ = 0.25; number of regions fixated per trial: *F*(2, 70) = 19.86, *p* < 0.001, *η*_p_^2^ = *η*_p_^2^ = 0.36). The same pattern held when analyzing the number of regions viewed per trial in the artificial foveation experiment. Once again there was a significant main effect of list order (*F*(2, 70) = 63.29, *p* < 0.001, *η*_p_^2^ = 0.64), a significant main effect of color coding (*F*(1, 70) = 92.77, *p* < 0.001, *η*_p_^2^ = 0.57), and a significant interaction between the two (*F*(2, 70) = 14.93, *p* < 0.001, *η*_p_^2^ = 0.30).

These results confirm that the differences in response times across list presentation conditions was driven by the length of the visual search process, rather than differences in the time required to come to a decision about each seal-container pair.

#### Refixations within trials

On each trial, we expected that the participants would identify an item to search for, then look for it in the other list while holding all or part of the target item in memory. When they found a potential match, they would move their eyes or their mouse back and forth between the lists to confirm that the items matched before making their response. Thus, we would expect multiple fixations and refixations on the item that the participant checked off on each trial and the corresponding item in the facility’s list, and fewer fixations on any items that were not the target on that particular trial. In addition, within each inspection list, there were different types of seal-container pairs that differed in difficulty. Items in the Match condition were relatively easy, as the participants simply had to confirm that an item in their inspection list and the matching item in the facility’s list were identical. However, checking off the other item types (Missing, Wrong Container, Transposed, and Transposed Match) was more complicated. The Missing items did not appear in the other list, so participants might check and recheck the whole list to confirm that an item was missing. The other items all had problems that could necessitate more comparisons back to the target item on the participant’s checklist. We expected to see more refixations of the target item and the corresponding item in the facility’s list for these conditions than for the Match condition.

**Fig. 7:**
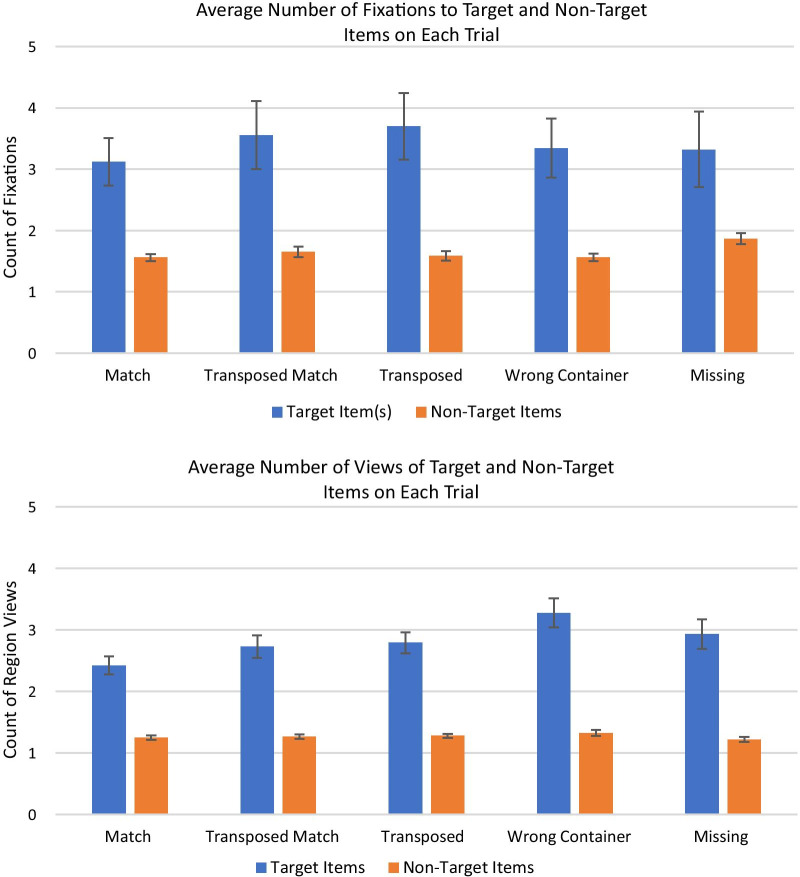
The average number of fixations to target items per trial for the eye tracking experiment (top) and the artificial foveation experiment (bottom). Error bars show the standard error of the mean

For this analysis, we focused only on the Random Order, No Color list presentation condition, because it was the most difficult condition and had the highest average number of fixations per trial. For each trial, we identified the item that the participant checked off on the checklist and its partner on the facility’s list as the “target items.” On trials where the participant checked off a Missing item, there was only one target item, but for every other trial type there were two target items, one on each list. We calculated the number of fixations to the target items on each trial, as well as the number of fixations to all non-target items that were fixated at least once. The average number of fixations to target and non-target items per trial is shown in Fig. 7.

For the eye tracking data, a 2 × 5 repeated measures ANOVA (target vs non-target by trial type) showed that there were significantly more fixations to targets than to non-targets (*F*(1, 126) = 86.21, *p* < 0.001, *η*_p_^2^ = 0.41), but there was not a main effect of trial type (Match, Missing, etc.; *F*(4, 126) = 0.37), nor was there a significant interaction (*F*(4, 126) = 0.49).

For the artificial foveation data, a 2 × 5 repeated measures ANOVA showed that there were significantly more views of regions containing targets than regions containing non-targets (*F*(4, 126) = 311.94, *p* < 0.001, *η*_p_^2^ = 0.71). There was also a significant main effect of trial type (*F*(4, 126) = 3.11, *p* < 0.02, *η*_p_^2^ = 0.09) but there was not a significant interaction (*F*(4, 126) = 2.11, *p* = 0.08). Pairwise comparisons using the Bonferroni Method showed that there were no significant differences in the number of views to non-target items across trial types. For the target items, the Match items had the lowest numerical number of views, but it was not significantly lower than the number of views to targets in the Transposed Match, Transposed, and Missing trials. However, the number of views to Match targets was significantly lower than the number of views to Wrong Container targets.

This analysis indicates that participants did look back and forth between the target items on both lists (and referred back repeatedly to the Missing items that appeared only on their checklist). We observed the same pattern in both the eye tracking and artificial foveation data. However, there were not substantial differences in the number of fixations to different types of targets. We predicted that the items containing errors would require more comparisons across lists than the Match items. While the Match items had the lowest average number of fixations per trial in both experiments, it was not significantly different from any of the other trial types in the eye tracking experiment, or from any trial type other than the Wrong Container items in the artificial foveation experiment.

#### Dwell times

**Fig. 8:**
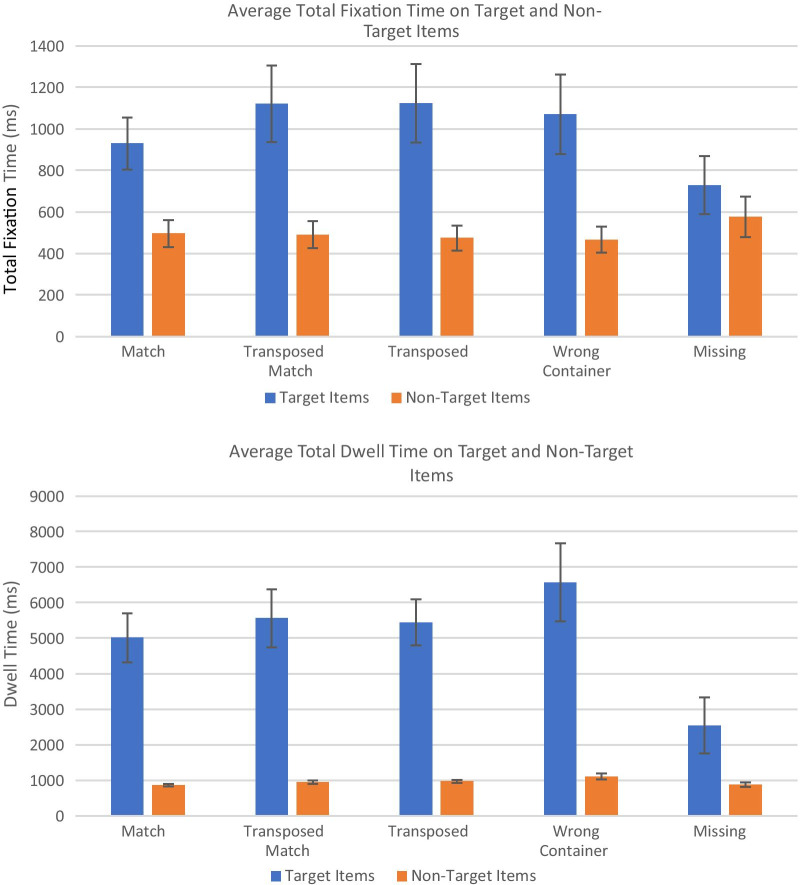
The average total fixation time on the target item(s) for each trial for the eye tracking experiment (top) and total dwell time for the artificial foveation experiment (bottom). Error bars show the standard error of the mean

In addition to having more fixations to the target items on each trial, we predicted that the participants in both experiments would have longer fixation durations for the target items. We also predicted that participants’ average fixation durations would be shorter for the Match items than for the more difficult item types. Once again, we assessed this using the Random Order, No Color list presentation condition. We calculated the dwell time, the total time participants spent fixating on each item, then calculated the average dwell times for the target and non-target items that were fixated on every trial. The results, averaged across trials of each type and then averaged across participants, are shown in Fig. 8.

For the eye tracking data, a 2 × 5 repeated measures ANOVA showed that the participants had significantly longer dwell times for the targets than for the non-targets (*F*(1, 126) = 61.45, *p* < 0.001, *η*_p_^2^ = 0.33). However, there was not a main effect of trial type (*F*(4, 126) = 0.84) or a significant interaction between target and trial type (*F*(4, 126) = 2.20, *p* = 0.07). This confirmed our prediction that participants would spend more time looking at the items they had selected as the targets of their visual search process on each trial. To test our second prediction, that the dwell times would be longer for non-match targets than for match targets, we used a paired t-test to compare the average total dwell time to targets that were match items versus targets that were not. The *t* test showed that there was not a significant difference in how long participants dwelled on match and non-match targets (*t*(14) = 0.97).

The results were very similar for the artificial foveation data. The average dwell times were significantly longer for targets than for non-targets (*F*(1, 125) = 250.10, *p* < 0.001, *η*_p_^2^ = 0.67), but there was not a significant main effect of trial type (*F*(4, 125) = 1.00, *p* = 0.41) or a significant interaction (*F*(4, 125) = 0.54). A paired *t* test comparing the participants’ average dwell times for match targets versus non-match targets showed that there was not a significant difference between the two (*t*(14) = 1.44, *p* = 0.09).

While the general pattern of dwell times was very similar for the two experiments, the dwell times were much longer for the artificial foveation experiment than for the eye tracking experiment. There are two likely causes for this difference. First, in the artificial foveation task, participants had to remove the mask on an item before they could begin reading it. This adds extra time to each region view, as does the slower speed at which people moved the mouse relative to the speed at which they moved their eyes from one item to the next. Second, the eye tracking data is noisier. Some gaze points were not included in any fixation, particularly when the participants were scanning rapidly through the list, and some fixations fell in between items due to drift in the tracking. The duration of those fixations would not have been included in this calculation if the center of the fixation did not fall inside of the region of interest. In this sense, the artificial foveation data is cleaner, because we know precisely how long each item appeared on the screen, while calculating fixation durations and assigning fixations to the correct item introduces some error.

#### Scanpaths

**Fig. 9:**
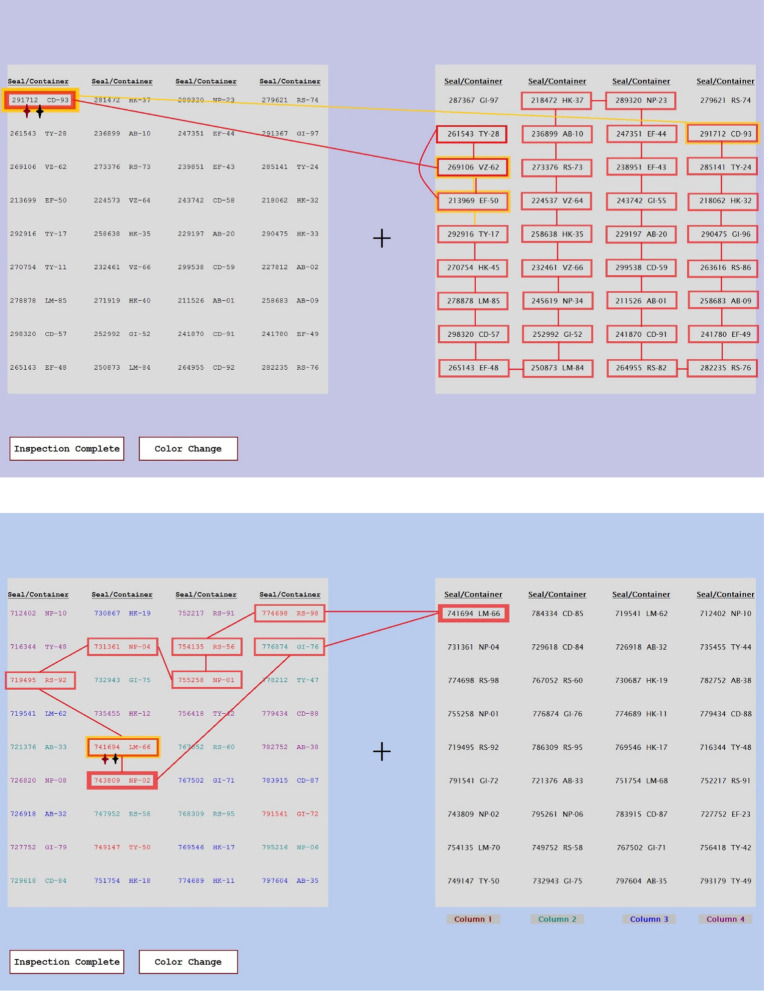
Examples of scanpaths obtained from the artificial foveation data. The top panel shows a trial from the Random Order, No Color condition and the bottom panel shows a trial from the Numerical Order, Color Coding condition. The first and last regions viewed are marked with red and black stars, respectively. The first visit to each region is indicated with a red box and the second visit is indicated with a yellow box. The box border is thicker when the dwell time in that region was more than 1 s

One of the key advantages of using eye tracking to study visual search is its ability to capture the path that participants take when scanning an image. As discussed in the introduction, scanpaths can provide insight into *how* participants are completing a task, and they can capture information that is difficult for participants to articulate when describing their own performance. We found the same to be true of the artificial foveation technique. We developed a visualization to show the order in which participants moused over each of the regions defined by the artificial foveation paradigm. Examples are shown in Fig. 9. The first region viewed during the trial is indicated with a red star and the last region viewed is indicated with a black star, mimicking the circles that are used to mark the beginning and end of the eye tracking data in the EyeWorks analysis software. The first visit to each region is represented by a red box surrounding that region. The border of the box is thin when participants spent less than one second viewing the region, and it is thick when participants dwelled on the region for more than one second. Lines connecting the boxes show the order in which the regions were viewed. Revisits to a region are depicted with a box of a different color placed around the first. Once again, this box has a thin border when the revisit lasted for less than one second, and a thick border when the revisit was longer than one second. It would be feasible to develop more nuanced representations of dwell time for each region, but this representation was sufficient for our purposes of performing qualitative assessments of the participants’ visual search strategies.

**Fig. 10:**
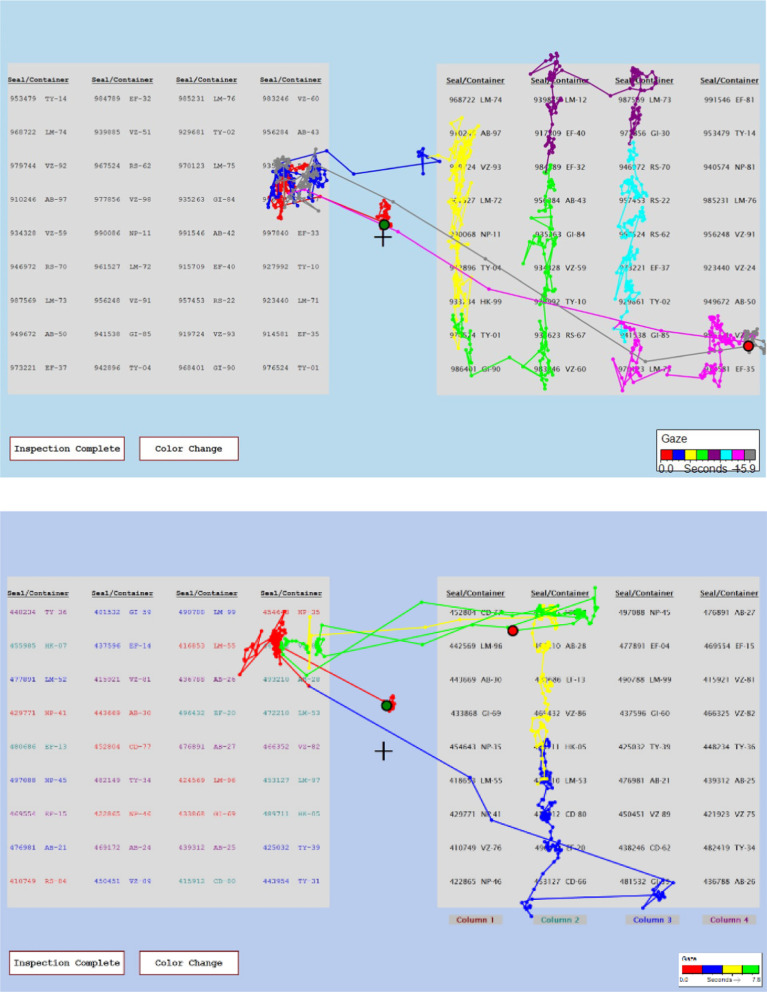
Examples of scanpaths obtained from the eye tracking data. The top panel shows a trial from the Random Order, No Color condition and the bottom panel shows a trial from the Random Order, Color Coding condition. The location of the participant’s gaze at the start and end of the trial is marked with green and red circles, respectively. The smaller circles indicate the gaze position each time the eye tracker sampled the data

One of the key questions in Gastelum et al. (2018) was whether participants would make use of the cues in the inspection list to constrain their visual search of the facility list. The faster response times for the color coding conditions imply that the participants were using the colors to constrain their search of the facility list, but the eye tracking information was needed to confirm this. Figure 10 shows eye tracking data from the Random Order, No Color condition (top) and from the Random Order, Color Coding condition (bottom). When no color coding was available, this participant searched up and down through all of the columns in the facility list before finding the target item. When color coding was available, as in the lower panel of Fig. 10, the participant’s visual search was constrained to the second column of the facility list, which was the column indicated by the color of the target item on the inspection checklist.

We observed very similar patterns for the artificial foveation scanpaths. The top panel in Fig. 9 shows a trial from the Random Order, No Color condition, just like the top panel of Fig. 10. Note that these two participants used search strategies that are nearly identical. Just like the eye tracking data, the artificial foveation data shows that the participant moved up and down the columns of the facility list until finding the target and then revisiting the initial region of interest to confirm that the two items matched. The lower panel of Fig. 9 shows a trial from the Numerical Order, Color Coding condition. In this case, the participant systematically checks all of the red items in the checklist when looking for a match for the first item on the facility list. Although this participant has a different search strategy than the participant whose eye tracking data are shown in the bottom panel of Fig. 10, the scanpath makes it clear that they were using the color cues to constrain their search.

## Discussion

In this study, we compared a standard eye tracking methodology to artificial foveation, an alternative method of assessing participants’ patterns of attention. The results revealed that both methods resulted in similar findings regarding the impact of list order and color coding on participants’ performance in a visual search task. In addition, both techniques revealed detailed information about how participants searched the lists on each trial.

At the block level (where each block consisted of checking off one list of 36 items), participants were faster and had fewer fixations or region views per trial when their checklist incorporated cues, such as color coding or helpful list ordering, that constrained their visual search process. An analysis of the participants’ response times for trials with different search set sizes found a roughly linear relationship between set size and response time, with a shallow slope that is similar to the slope observed in classic visual search tasks that require inefficient visual search, such as the T and L task (Wolfe, 1998). We found overall slower response times for the artificial foveation procedure due to the speed limitations imposed by moving the mouse instead of simply moving one’s eyes. However, the constraints introduced by the artificial foveation procedure did not change the participants’ patterns of performance across conditions or the dynamics of their visual search process. We found similar patterns of results for the two data collection methodologies across all of our behavioral and eye tracking-based metrics.

At the trial level, participants in the eye tracking and artificial foveation experiments generally used similar task completion strategies. The participants in both experiments selected a target from their checklist and searched for it in the facility’s list, scanning through the items until they found a likely match. Then they compared the probable match back to the target on their checklist in order to identify any discrepancies between the two. This led to participants having many more fixations or views to the target and its partner than to any of the distractor items (all items that were not the target of the participant’s search on that trial). While we predicted that participants would spend more time looking at the target items in conditions where there was an error on the facility’s list (Transposed, Wrong Container, or Missing items), we generally did not see substantial differences between the different item types. This indicates that participants made roughly the same number of comparisons between the target item and its probable match on the facility’s list when the two items matched exactly and when the two items had discrepancies.

This experiment differs from many classic visual search studies because the stimuli on the screen remained the same throughout the 36 trials in each block, but the participant’s choice of a search target on each trial changed which items were targets and which were distractors. Our choice of task was driven by the real-world constraints faced by international nuclear safeguards inspectors, who must compare detailed records from prior inspections to a nuclear facility’s current inventory. While the inspectors have no control over the facility’s inventory records and how they are formatted, it is possible for them to format their records from prior inspections in ways that make this tedious task faster and easier. For example, they could place the items in their own checklists in numerical order, or they could color code them based on shipment date, storage location, or any other information that would constrain their search through the facility’s records. Thus, in these experiments, we manipulated the list order and color coding to test which types of cues were most useful to the participants. In the Random Order, No Color condition, which is most representative of the real-world inspection task, the participants had to search through all 36 items on the facility’s list until finding their target. They repeated this process on every trial, in a slow and tedious inspection. At the other extreme, in the Facility Order conditions, the participants simply had to compare the items in the same position on the two lists. The effective set size for their visual search process was one item, so they were able to complete each trial very quickly. While matching the orders of the two lists under comparison is clearly ideal, it is unlikely to be feasible in real facility inspections. However, color coding and/or placing the inspection checklist in numerical order are both feasible ways of constraining the inspectors’ search processes. In these experiments, we found that the participants were able to make good use of the color coding cues. The participants’ scanpaths and response times showed that they used the color cues to constrain their visual search to the appropriate column of the facility’s list. On the other hand, very few participants made use of the numerical ordering. Only four participants in each experiment reported that they used this strategy, and none of them used it when they could use the color cues instead. This indicates that numerical ordering, while simple to implement on an inspector’s checklist, may not be helpful to the inspectors unless they are trained on how to change their search strategy to take advantage of this information.

### Limitations

The fact that we did not use a classic visual search task leaves some questions about the similarities and differences between eye tracking and artificial foveation unanswered. For example, we do not know how changing the size of the stimuli or changing the size of the artificial foveation window might impact this comparison. In our task, the alphanumeric strings were small enough that participants had to fixate on them in order to read them. In a task that used larger stimuli or targets that could be identified using peripheral vision, we might see more differences between data collected via eye tracking and data collected via mouse tracking or other artificial foveation techniques. In that case, participants might struggle more with the fact that portions of the image are obscured and might adopt different strategies to compensate for that difficulty.

We also do not know how changes to the size of the artificial foveation window would impact visual search performance. In the current study, we simply sized the windows so that each showed one seal-container pair, but the window could be larger (multiple items revealed at once) or smaller (part of an item). Smaller windows would provide more granular information. In this case, smaller windows would have allowed us to test whether participants were searching based on the seal number or the container number. Using smaller windows would likely have produced a closer match between the average number of fixations to target items and the average number of region views in the artificial foveation experiment. The seal-container pairs were long enough that multiple fixations were required to read the entire string. In the eye tracking data, those fixations are counted separately, but in the artificial foveation experiment a single region view could encompass multiple fixations. In a study with larger stimuli and larger windows, this difference would be even more pronounced. On the other hand, larger windows might reduce average response times, bringing them more in line with the response times obtained in the eye tracking paradigm. Similar manipulations of window size have been explored in the gaze-contingent reading literature (e.g., Rayner, 2014), but could also be tested in the context of classic visual search tasks. The appropriate window size is likely to depend on the task, the stimuli, and the research question of interest, given that the size of the perceptual span is smaller for reading than in scene perception and visual search (e.g., Rayner, 2009). Future research in this area could vary the size of the artificial foveation window to see if the size of the window impacts the participants’ response times or visual search behavior. Another fruitful avenue for future research in this domain would be testing the artificial foveation approach in the context of multiple target search, where different metrics regarding search strategy and search termination are of interest to researchers.

The slower pace of artificial foveation relative to eye tracking is also an important limitation. As shown in Fig. 4, it took roughly twice as long for participants to complete each trial in the artificial foveation experiment relative to the eye tracking experiment. This difference is likely due to the fact that moving the mouse is substantially slower than moving one’s eyes. On each trial, participants viewed multiple list items to find and compare the target items, and it took them longer to view each one when they had to move the mouse in order to reveal them. Anecdotally, the slower pace of the artificial foveation task could be frustrating for the participants. The additional time needed to collect the artificial foveation data is also important because the settings in which eye tracking is not feasible are often operational settings, such as secure buildings where recording devices like eye tracking cameras are not allowed. Researchers working in those settings often have limited time with their participants, so the fact that our implementation of artificial foveation slows down task completion could be problematic. The approach we used here is also less naturalistic than eye tracking. It requires participants to interact with images in a less natural way than by simply moving their eyes. It also requires a substantial amount of advance preparation to set up the experimental stimuli and the regions of interest for the artificial foveation technique. However, we would argue that a similar level of advance preparation is necessary for conducting well-controlled eye tracking experiments.

### Conclusions

Although mouse tracking is sometimes called “poor man’s eye-tracking” (Mancas & Ferrera, 2016), our study demonstrates that implementing an artificial foveation technique via mouse tracking can provide effective characterizations of participants’ visual search behavior. Moving the mouse to view different regions of the screen impacts performance by slowing response times, but the artificial foveation technique and traditional eye tracking methods can reveal the same patterns of visual search performance and strategy across conditions. Our findings align with prior research using free viewing tasks, which have indicated that artificial foveation can be an effective substitute for eye tracking when determining which regions of an image are viewed most often (Jiang et al., 2015; Kim et al., 2017).

A major advantage of artificial foveation is that this data can be collected outside of laboratory settings. This is particularly relevant in the context of the ongoing COVID-19 pandemic, which has made it difficult to conduct in-person human subjects experiments. Artificial foveation techniques can enable visual cognition researchers to continue their work via remote or online data collection. Participants can complete an artificial foveation task using their own computer, without having to travel to a lab with an eye tracker set up. There is no need for calibration or specialized equipment, so data can be collected without an experimenter present. Mouse tracking can also be implemented in platforms such as Amazon Mechanical Turk, and prior research has indicated that collecting mouse tracking data online produces very similar results to collecting mouse tracking data in the laboratory (Jiang et al., 2015).

Overall, our findings indicate that artificial foveation is a useful tool for studying visual search in situations where eye tracking is not feasible. While there are some drawbacks to this technique, there are cases in which it provides valuable information that cannot be obtained in other ways.

## Supplementary Information

**Additional file 1.** Example of artificial foveation technique implemented in E-Prime 3.0.

## Data Availability

The datasets and code used in these experiments are available from the corresponding author upon request.
