# Supplementary figures and images for: Studying visual search without an eye tracker: an assessment of artificial foveation
Source: Cogn Res Princ Implic. 2021 Jun 25;6:45. doi: 10.1186/s41235-021-00304-2 (PMC8226349; doi:10.1186/s41235-021-00304-2)

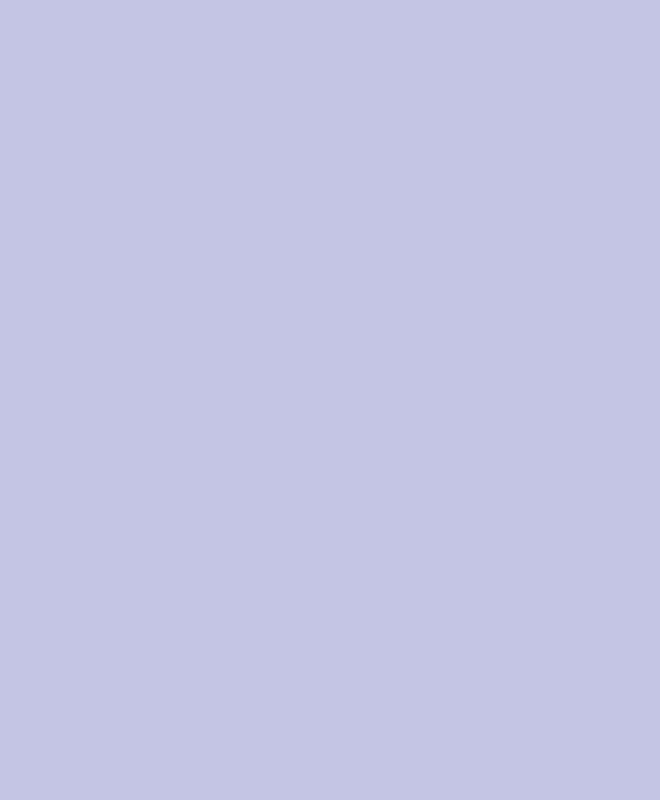

Supplement: Supplementary file 1 — Additional file 1. Example of artificial foveation technique implemented in E-Prime 3.0. [file 41235_2021_304_MOESM1_ESM.zip › ArtificialFoveationExample/BackgroundA.jpg]

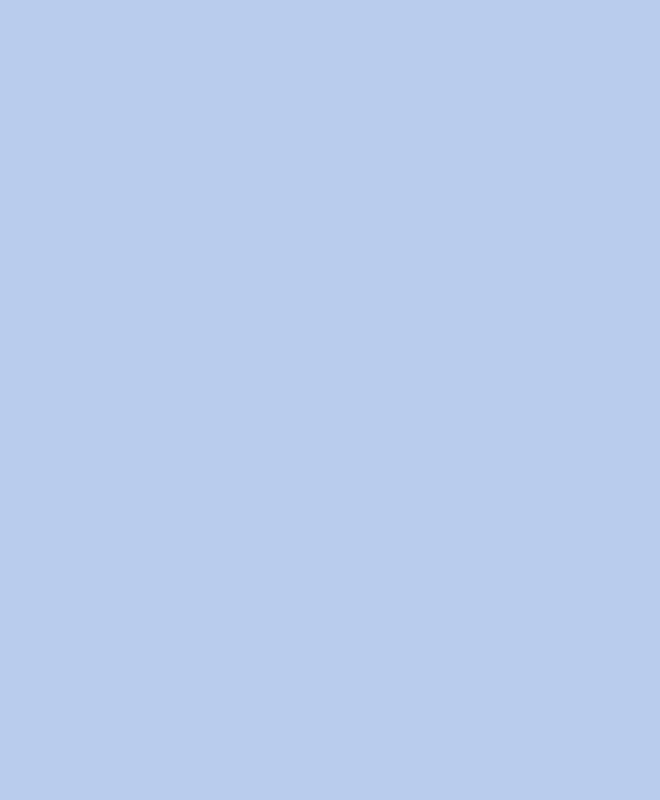

Supplement: Supplementary file 1 — Additional file 1. Example of artificial foveation technique implemented in E-Prime 3.0. [file 41235_2021_304_MOESM1_ESM.zip › ArtificialFoveationExample/BackgroundB.jpg]

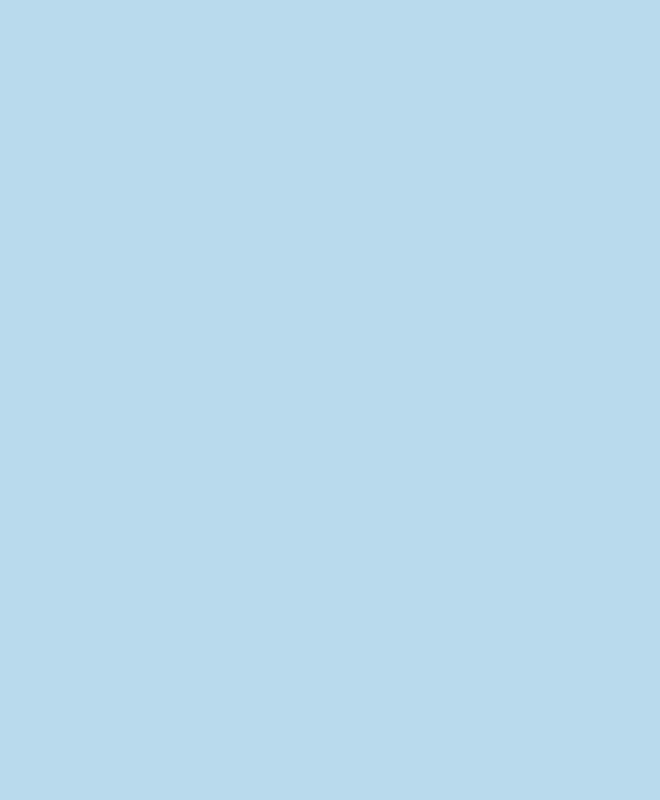

Supplement: Supplementary file 1 — Additional file 1. Example of artificial foveation technique implemented in E-Prime 3.0. [file 41235_2021_304_MOESM1_ESM.zip › ArtificialFoveationExample/BackgroundC.jpg]

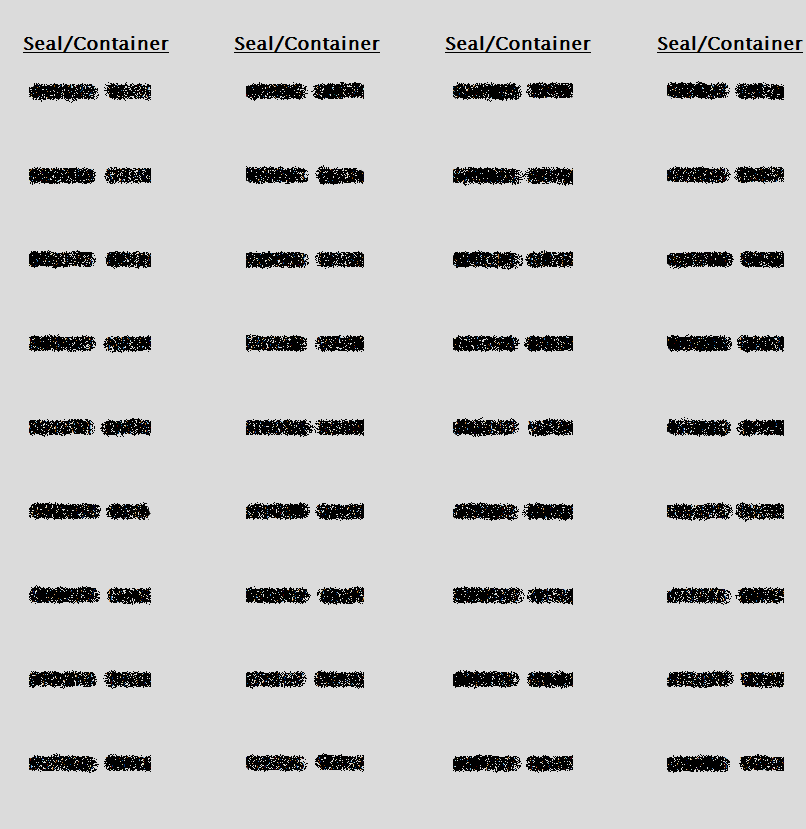

Supplement: Supplementary file 1 — Additional file 1. Example of artificial foveation technique implemented in E-Prime 3.0. [file 41235_2021_304_MOESM1_ESM.zip › ArtificialFoveationExample/FacListblur4.bmp]

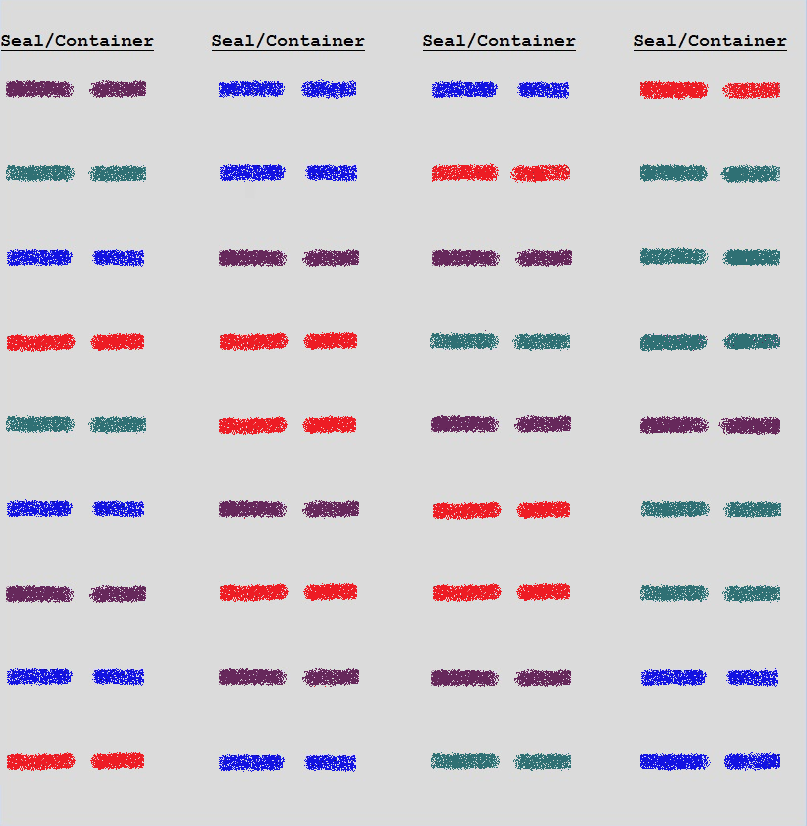

Supplement: Supplementary file 1 — Additional file 1. Example of artificial foveation technique implemented in E-Prime 3.0. [file 41235_2021_304_MOESM1_ESM.zip › ArtificialFoveationExample/RandFacBack2.bmp]
